# Pharmacological characterization of P2Y receptor subtypes – an update

**DOI:** 10.1007/s11302-023-09963-w

**Published:** 2023-09-12

**Authors:** Ivar von Kügelgen

**Affiliations:** Department of Pharmacology and Toxicology, Pharma Center, University of Bonn, D-53127 Bonn, Germany

**Keywords:** P2Y receptors, Agonists, Antagonists, Labelled ligands

## Abstract

P2Y receptors are G-protein-coupled receptors (GPCRs) for extracellular nucleotides. There are eight mammalian P2Y receptor subtypes (P2Y_1_, P2Y_2_, P2Y_4_, P2Y_6_, P2Y_11_, P2Y_12_, P2Y_13_, and P2Y_14_). The widely expressed P2Y receptors play important roles in physiology and pathophysiology. This review summarizes the use of pharmacological tools to characterize the P2Y receptor subtypes involved in these responses. MRS2500 is a potent and selective antagonist acting at the P2Y_1_ receptor. AR-C118925 is useful for the selective antagonism of the P2Y_2_ receptor. PSB16133 blocks the P2Y_4_ receptor, MRS2578 is an antagonist at the P2Y_6_ receptor and NF157 as well as NF340 block the P2Y_11_ receptor. ADP-induced platelet aggregation is mediated by P2Y_1_ and P2Y_12_ receptors. A number of compounds or their active metabolites reduce ADP-induced platelet aggregation by blocking the P2Y_12_ receptor. These include the active metabolites of the thienopyridine compounds clopidogrel and prasugrel, the nucleoside analogue ticagrelor and the nucleotide analogue cangrelor. PSB0739 is also a potent antagonist at the P2Y_12_ receptor useful for both in vitro and in vivo studies. MRS2211 and MRS2603 inhibit P2Y_13_ mediated responses. PPTN is a very potent antagonist at the P2Y_14_ receptor.

This article is dedicated to late Professor María Teresa Miras-Portugal. She was a leading scientist in the field of purinergic signaling and P2 receptors for extracellular nucleotides [111–114]. She contributed to many important studies, advisory and editorial boards, the Purine club and the IUPHAR sub-committee for the nomenclature of P2Y receptors. She was the expert in the field of biochemistry, physiology and pharmacology of dinucleoside polyphosphates.

There are eight human subtypes of G-protein-coupled receptors (GPCRs) for extracellular nucleotides, P2Y receptors [23, 89]. They belong to the delta-subgroup of class A GPCRs [49]. The P2Y receptor family can be divided into two subfamilies [2, 156]. The first subfamily consists of the P2Y_1_, P2Y_2_, P2Y_4_, P2Y_6_ and P2Y_11_ receptors. The receptors couple via G_q_-proteins to stimulation of phospholipase C (references in Tables 1, 2, 3, 4, and 5). P2Y_11_-receptors couple in addition through G_s_ and increases in adenylate cyclase activity (references in Table 5). In contrast, the P2Y_12_ receptor subfamily (P2Y_12_, P2Y_13_, and P2Y_14_ receptors) signals through activation of G_i_-proteins (references in Tables 6, 7, and 8; see also [83, 158, 160]. P2Y receptors play important roles in physiology and pathophysiology [2, 22, 24, 120, 138, 139, 156, 158, 159]. Genetic knockout models can be used to identify the subtypes involved in responses to nucelotides. A fast alternative is the use of subtype specific ligands modifying cellular or tissue responses. Due to the efforts of medicinal chemistry novel compounds have been developed [10, 80–82, 118, 119, 121, 135, 143]. This article discusses pharmacological tools used to characterize P2Y receptor subtypes.

**Table 1:** Selected ligands acting at the human P2Y_1_-receptor

		pEC_50_/pK_i_	Selected references
Principal agonist	ADP	6.0–7.0	[161, 162]
Synthetic agonist	2-methylthio-ADP	6.0–7.0	[161, 162]
Selective agonist	MRS2365	9.4	[30]
Selective antagonist	MRS2500	9.1	[91, 171]
Radioligand	[^32^P]MRS2500	9.0	[73]
Allosteric antagonist	BPTU	8.2	[28, 53, 171]

Modified from von Kügelgen and Hoffmann [159], von Kügelgen ([157, 160]) and Jacobson et al. [83]. BPTU, 1-(2-(2-(tert-butyl)phenoxy)pyridin-3-yl)-3-(4-(trifluoromethoxy)phenyl)urea; MRS2365, (N)-methanocarba-2-methylthio-ADP; MRS2500, 2-iodo-N6-methyl-(N)-methanocarba-2´-deoxyadenosine 3´,5´-bisphosphate

**Table 2:** Selected ligands acting at the human P2Y_2_-receptor

		pEC_50_/pK_i_	Selected references
Principal agonist	UTP	8.0	[96, 102]
	ATP	7.0	[96, 102]
	Ap4A	6.1	[96]
Agonist	Diquafosol	7.0	[131]
Selective agonist	PSB-1114	7.3	[43]
	MRS2698	8.1	[77]
Antagonist	PSB-416	4.7	[66]
Selective antagonist	AR-C118925	7.2	[87, 92, 136]
Fluorescent ligand	of AR-C118925	6.3	[37]

Modified from von Kügelgen and Hoffmann [159], von Kügelgen ([157, 160]) and Jacobson et al. [83]. Ap4A, diadenosine-tetraphosphate; AR-C118925, 5-[[5-(2,8-dimethyl-5H-dibenzo[a,d]cyclohepten-5-yl)-3,4-dihydro-2-oxo-4thioxo-1(2H)-pyrimidinyl]methyl-N-2H-tetrazol-5-yl-2-furancarboxide; MRS2698, 2'-amino-2'-deoxy-2-thio-UTP; PSB-416, 1-amino-4-(4-methoxyanilino)-9,10-dioxoanthracene-2-sulfonic acid; PSB-1114, 4-thiouridine-5´-O-(β,γ-difluoromethylene)triphosphate

**Table 3:** Selected ligands acting at the human P2Y_4_-receptor

		pEC_50_/pK_i_	Selected references
Principal agonist	UTP	6.3	[32, 122, 123]
Selective agonist	MRS4062	7.6	[108]
Antagonist	ATP	6.2	[88]
Selective inhibitor	PSB-1699	6.4	[137]
	PSB-16133	6.6	[137]

Modified from von Kügelgen and Hoffmann [159], von Kügelgen ([157, 160]) and Jacobson et al. [83]. MRS4062, N4-(phenylpropoxy)-CTP; PSB-1699, 1-amino-4-[4-(3-pyridin-3-ylmethylthio)phenylamino]-9,10-dioxo-9,10-dihydroanthracene-2-sulfonate; PSB-16133, 1-amino-4-[4-(2,4-dimethylphenylthio)phenylamino]-9,10-dioxo-9,10-dihydroanthracene-2-sulfonate

**Table 4:** Selected ligands acting at the human P2Y_6_-receptor

		pEC_50_/pK_i_	Selected references
Principal agonist	UDP	6.5	[33, 123]
Selective agonist	MRS2693	7.8	[17]
	MRS2957	7.9	[107]
	5-OMe-UDP(α-B)	8.1	[59, 61]
Selective antagonist	MRS2578	7.4	[104]

Modified from von Kügelgen and Hoffmann [159], von Kügelgen ([157, 160]) and Jacobson et al. [83]. MRS2578, N,N''-1,4-butanediylbis[N'-(3-isothiocyanatophenyl)thiourea; MRS2693, 5-iodo-UDP; MRS2957, P1-[5'(N4-Methoxycytidyl)]-P3-(5'-uridyl)-triphosphate; 5-OMe-UDP(α-B), 5-OMe-uridine-5′-O-(α-boranodiphosphate)

**Table 5:** Selected ligands acting at the human P2Y_11_-receptor

		pEC_50_/pK_i_	Selected references
Principal agonist	ATP	4.2–4.7	[34, 35], Qi et al. 2001
Synthetic agonist	NF546	6.3	[109]
	AR-C67085	5.5	[35]
Selective antagonist	NF157	7.3	[154]
	NF340	8.0	[109]

Modified from von Kügelgen and Hoffmann [159], von Kügelgen ([157, 160]) and Jacobson et al. [83]. AR-C67085, 2-propylthio-β,γ-difluoromethylene-D-ATP; NF157, 8,8'-[carbonylbis[imino-3,1-phenylenecarbonylimino(4-fluoro-3,1-phenylene)carbonylimino]]bis-1,3,5-naphthalenetrisulfonic acid; NF340, 4,4´-(carbonylbis(imino-3,1-(4-methyl-phenylene)carbonylimino))bis(naphthalene-2,6-disulfonic acid); NF546, 4,4´-(carbonylbis(imino-3,1-phenylene-carbonylimino-3,1-(4-methyl-phenylene)carbonylimino))-bis(1,3-xylene-α,α´-diphosphonic acid)

**Table 6:** Selected ligands acting at the human P2Y_12_-receptor

		pEC_50_/pK_i_	Selected references
Principal agonist	ADP	6.0	[47, 70, 152]
Synthetic agonist	2-methylthio-ADP	9.0	[152]
Antagonist	Cangrelor	9.4	[75]
	AR-C67085	8.2	[75]
Selective antagonist	Ticagrelor	8.6	[68, 146]
	Selatogrel	7.4	[9]
	PSB-0739	9.8	[11]: [67]
Irreversible inhibitor	Clopidogrel (active metabolite)	6.9	[65]
Radioligand	[^3^H]2-methylthio-ADP	9.0	[152]
	[^3^H]PSB0413	8.3	[42, 125]

Modified from von Kügelgen and Hoffmann [159], von Kügelgen ([157, 160]) and Jacobson et al. [83]. AR-C67085, 2-propylthio-β,γ-difluoromethylene-D-ATP; [^3^H]PSB0413, [^3^H]2-propylthioadenosine-5'-adenylic acid (1,1-dichloro-1-phosphonomethyl-1-phosphonyl) anhydride; PSB-0739, 1-amino-9,10-dihydro-9,10-dioxo-4-[[4-(phenylamino)-3-sulfophenyl]amino]-2-anthracenesulfonic acid

**Table 7:** Selected ligands acting at the human P2Y_13_-receptor

		pEC_50_/pK_i_	Selected references
Principal agonist	ADP	6.5	[36, 106]
Synthetic agonist	2-methylthio-ADP	9.0	[106]
Antagonist	Cangrelor	8.3	[106]
Selective antagonist	MRS2211	6.0	[90]
	MRS2603	6.2	[90]
Radioligand	[^32^P]2-methylthio-ADP 9.6	[106]	

Modified from von Kügelgen and Hoffmann [159], von Kügelgen ([157, 160]) and Jacobson et al. [83]. MRS2211, 2-[(2-chloro-5-nitrophenyl)azo]-5-hydroxy-6-methyl-3-[(phosphonooxy)methyl]-4-pyridinecarboxaldehyde; MRS2603, [(2Z)-2-[(4-chloro-3-nitrophenyl)hydrazinylidene]-4-formyl-6-methyl-5-oxopyridin-3-yl]methyl dihydrogen phosphate

**Table 8:** Selected ligands acting at the human P2Y_14_-receptor

		pEC_50_/pK_i_	Selected references
Principal agonist	UDP	6.5	[51]
	UDP-glucose	7.6	[27, 29, 50]
Selective agonist	2-thio-UDP	8.7	[40]
	MRS2905	9.0	[40]
Selective antagonist	MRS4608	7.7	[85]
	PPTN	10.1	[13]
Fluorescent antagonist	MRS4183	9.0	[93]

Modified from von Kügelgen and Hoffmann [159], von Kügelgen ([157, 160]) and Jacobson et al. [83]. MRS2905, α,β-methylene-2-thio-UDP; phenylpropoxy)-CTP; MRS4183, 4-((piperidin-4-yl)-phenyl)-7-(4-(trifluoromethyl)-phenyl)-2-naphthoic acid-Alexa Fluor 488 conjugate; MRS4608, 4-(4-(quinuclidin-4-yl)phenyl)-7-(4-(trifluoromethyl)phenyl)-2-naphthoic acid; PPTN, 4-((piperidin-4-yl)-phenyl)-7-(4-(trifluoromethyl)-phenyl)-2-naphthoic acid

When using nucleotides such as ATP as agonists an interaction with ecto-nucleotidases [172] may be important. Hence, ATP may be degraded to ADP and further to adenosine which may activate adenosine receptors [74].

## P2Y_1_ receptor ligands

The P2Y_1_ receptor plays important roles in physiology including vasodilation, platelet aggregation, pain sensation, and astroglial signaling [2, 12, 48, 78, 124]. ADP is the principal agonist acting at the human P2Y_1_ receptor Table 1, [7, 84, 98]. Application of ATP may induce responses mediated by the P2Y_1_ receptor due to the fast breakdown of ATP to ADP [172]. When studied in detail with purified nucleotides, ATP has also been shown to act as a partial agonist at the receptor [64, 161]. 2-Methylthio-ADP activates the receptor (Table 1). The Northern (N-methanocarba analogue of 2-methylthio-ADP MRS2365; Table 1; [30] is a very potent and selective agonist that is inactive at the other two ADP receptors, P2Y_12_ and P2Y_13_.

Bisphosphate analogues including MRS2279 (2-chloro-N^6^-methyl-(N)-methanocarba-2´-deoxyadenosine 3´,5´-bisphosphate; [162] and MRS2500 (2-iodo-N^6^-methyl-(N)-methanocarba-2´-deoxyadenosine 3´,5´-bisphosphate; [91] act as antagonists. MRS2500 is a potent and selective antagonist Table 1, [91]. 2,2´-Pyridylisatogen tosylate [54] and BPTU (N-[2-[2-(1,1-dimethylethylphenoxy]-3-pyridinyl]-N'-[4-(trifluoromethoxyphenyl]urea; [28, 171] act as allosteric inhibitors. Interestingly, MRS2500 and BPTU have been used to grow crystals for the crystallography of the human P2Y_1_ receptor protein [171]. The data of that study clearly show two disparate ligand-binding sites at the receptor protein [171]. MRS2500 binds within the seven transmembrane helixes and BPTU binds to an allosteric pocket on the receptor interface with the cell membrane [171].

([^32^P]-labeled and [^125^I]-labeled) analogues of MRS2500 can be used for binding studies [73, 124], Table 1. A [^18^F]PET tracer ([^18^F]1-{2-[2-(tert-butyl)phenoxy]pyridin-3-yl}-3-[4-(2-fluoroethyl)phenyl]urea) is available for imaging of the tissue distribution of P2Y_1_ receptors [116].

## P2Y_2_ receptor ligands

The P2Y_2_ receptor plays roles in ion transport, regulation of epithelial cells, migration, vasodilatation and immune responses [31, 44, 97, 133, 140, 141]. Both ATP and UTP activate the receptor Table 2, [45]. Diadenosine-tetraphosphate (Ap4A), Up4U (diquafosol, INS365 [96, 131], and P^1^-(uridine 5´)-P^4^-(2´-deoxycytidine-5´)tetraphosphate (INS37217, denufosol; [83] also act as agonists. In fact, diquafosol is used in Japan and South Korea for the treatment of the dry eye syndrome [153, 165]. The 2-thio-analogues of UTP MRS2698 and PSB-1114 are more selective P2Y_2_ receptor agonists Table 2, [43, 77].

AR-C118925 is a potent and selective non-nucleotide antagonist at the P2Y_2_ receptor Table 2; [87, 92, 136, 137]. A fluorescent analogue of AR-C118925 can be used for labeling of the receptor at the cell membrane [37].

## P2Y_4_ receptor ligands

The P2Y_4_ receptor controls cellular responses including ion transport, growth and migration [71, 142]. The human P2Y_4_ receptor is activated by UTP and blocked by ATP Table 3, [32, 88, 123]. In contrast, both UTP and ATP act as full agonists at the rat P2Y_4_ receptor [21]. CTP analogues including MRS4062, and N^4^-(phenylethoxy)-CTP activate the human P2Y_4_ receptor with a preference over P2Y_2_ and P2Y_6_ receptors Table 3, [108].

PSB-1699 and PSB-16133 inhibit P2Y_4_ receptor activation with a clear selectivity versus other P2Y receptor subtypes [137]. They may act as allosteric inhibitors [137].

## P2Y_6_ receptor ligands

The human P2Y_6_ receptor plays roles vasoconstriction, ion secretion, migration, and inflammation [58, 60, 95, 97, 100, 103, 115, 134]. UDP is the endogenous agonist activating the receptor Table 4, [33, 123]. Synthetic agonists include PSB-0474 [43], MRS2693 [17] and the R(p) isomer of 5-OMe-UDP(α-B) Table 4, [59, 79].

MRS2578 blocks the P2Y_6_ receptor in a non-surmountable manner Table 4; [104]. MRS2578 may exert off target effects inhibiting cell migration [145]. TIM-38 (3-nitro-2-(trifluoromethyl)-2H-chromene) is a novel antagonist inhibiting P2Y_6_ receptor activation with an IC_50_ value of 4.3 μM and selectivity versus other P2Y receptor subtypes [76].

## P2Y_11_ receptor ligands

The P2Y_11_ receptor plays roles in granulocyte differentiation, dendritic cell maturation, chemotactic responses of neutrophils and neuropathic pain [164]. The human P2Y_11_ receptor is activated by ATP Table 5, [34]. In contrast, ADP is the principal agonist activating the canine P2Y_11_ receptor [166]. Synthetic agonists include the P2Y_12_ receptor antagonist 2-propylthio-β,γ-dichloromethylene-D-ATP (AR-C67085) and the suramin analogue NF546 Table 5, [109].

Suramin and the suramin analogues NF157 and NF340 act as antagonists at the human P2Y_11_ receptor Table 5; [109, 154]. NF340 is selective and slightly more potent Table 5, [109].

## P2Y_12_ receptor ligands

The P2Y_12_ receptor plays a crucial role in ADP-induced platelet aggregation [26, 63, 70, 152, 168]. Receptor activation is also involved in vasoconstriction, control of microglia, immune responses and control of bone mass [5, 16, 62, 72, 105, 115, 148, 151, 163]. As mentioned above, ADP is the endogenous agonist acting at the P2Y_12_ receptor Table 6, [30, 70]. ATP has antagonistic properties [86]. 2-Methylthio-ADP is a more potent agonist (Table 6).

Reduction of platelet aggregation is an important strategy in the prevention or therapy of cardiovascular events such as myocardial infarction. A number of potent antagonists have been developed (Table 6). AR-C67085 and cangrelor (AR-C69931MX; [75] are surmountable antagonists. Cangrelor is used to reduce platelet aggregation by intravenous application [99]. Cangrelor also blocks the P2Y_13_ receptor in a non-competitive manner see below Table 7, [106].

The radiolabeled nucleotide derivative [^3^H]PSB-0413 has a nanomolar affinity at the P2Y_12_ receptor Table 6; [42, 125]. The nucleoside analogue ticagrelor (AZD6140) is orally available and used for the reduction of platelet aggregation in patients after myocardial infarction [126, 155]. Ticagrelor appears to act in a surmountable manner at the human P2Y_12_ receptor Table 6, [68], see also [6, 57, 129]. In addition to the P2Y_12_ receptor, ticagrelor also blocks the P2Y_13_ receptor [19] and the equilibrative nucleoside transporter 1 [6]. Non-nucleotide antagonists include the analogue of reactive blue 2, PSB-0739 Table 6, [11, 67], 6-amino-2-thio-3H-pyrimidin-4-one derivatives [38], ethyl 6-aminonicotinate acyl sulfonamides [8], flavonolignans [18], morpholine derivatives [3], piperazinyl glutamates [130, 167], as well as salvianolic acids [101]. Selatogrel (ACT-246475) has a nanomolar affinity at the human P2Y_12_ receptor [9] and can be applied by subcutaneous administration [14, 147]. Selatogrel may act as inverse agonist at the human P2Y_12_ receptor [132].

**Fig. 1:**
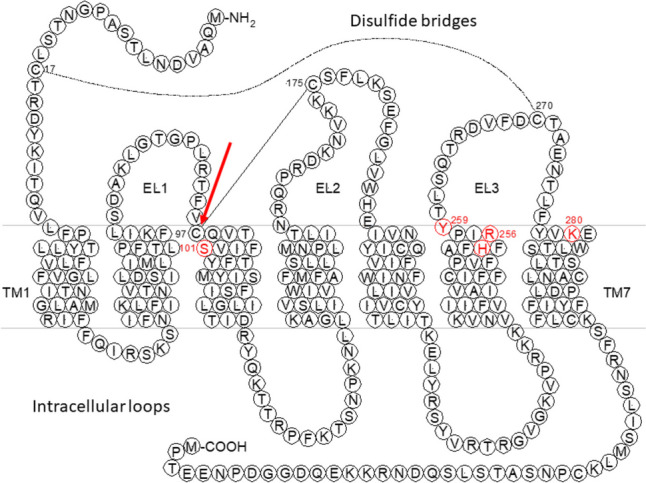
Predicted two-dimensional structure of the human P2Y_12_ receptor. TM transmembrane region, EL extracellular loop. Modified from [156]. The red arrow indicates Cys97 that is important for the interaction with the active metabolites of clopidogrel and prasugrel [4, 41, 144] and plays a role in receptor activation [169, 170]. The roles of the amino acid residues marked in red have been analyzed by Hoffmann et al. [69]. Arg256 and Lys280 are important for ligand recognition.

Thienopyridine compounds are used for decades to reduce platelet aggregation in patients with cardiovascular diseases [15, 26]. The active metabolites of clopidogrel Table 6, [144], and prasugrel [149, 150] interact in an irreversible manner with Cys97^3.25^ of the receptor protein (see red arrow in Fig. 1, [4, 41, 144]. Treatment with prasugrel is more potent when compared to ticlopidine or clopidogrel [15].

The crystallography of P2Y_12_ receptor proteins bound to agonists and antagonists will further facilitate the development of novel P2Y_12_ receptor ligands [169, 170].

## P2Y_13_ receptor ligands

The P2Y_13_ receptor is involved in degranulation of mast cells, metabolic effects and neuroprotection [20, 46, 55, 127, 128]. ADP is the principal agonist activating the P2Y_13_ receptor Table 7, [36]. ATP may act as a partial agonist [106].

Analogues of PPADS (pyridoxalphosphate-6-azophenyl-2',4'-disulfonic acid) including MRS2211 and MRS2603 inhibit the activation of the human P2Y_13_ receptor Table 7; [90]. Cangrelor blocks both P2Y_12_ receptor and the P2Y_13_ receptor [52, 106].

## P2Y_14_ receptor ligands

The P2Y_14_ receptor is involved in inflammation, pain, control of mast cells and microglia, insulin release, and vasoconstriction [1, 13, 39, 56, 110, 117]. UDP and UDP-sugars such as UDP-galactose and UDP-glucose activate the receptor Table 8, [25, 27, 50]. UDP analogues such as 5-iodo-UDP (MRS2690) and α,β-methylene-2-thio-UDP (MRS2905) are potent and selective agonists activating the P2Y_14_ receptor Table 8, [25, 40, 94].

Potent and selective antagonists include MRS4608 Table 8; [85] and PPTN (4-[4-(4-piperidinyl)phenyl]-7-[4-(trifluoromethyl)phenyl]-2-naphthalenecarboxylic acid; Table 8; [13]. MRS4174 is a fluorescent antagonist with an affinity in the nanomolar range Table 8, [93].

## Data Availability

“Not applicable”.
